# A LIFE COURSE APPROACH TO NEIGHBORHOOD SEGREGATION AND HEALTH AMONG BLACK AND WHITE AMERICANS

**DOI:** 10.1093/geroni/igae098.2866

**Published:** 2024-12-31

**Authors:** Kayla Fike, Huaman Sun, Michael Esposito, Dominique Sylvers, Kate Duchowny, Hedwig Lee, Margaret Hicken

**Affiliations:** Vanderbilt University, Nashville, Tennessee, United States; University of Toronto, Toronto, Ontario, Canada; University of Minnesota, Minneapolis, Minnesota, United States; University of Michigan, Ann Arbor, Michigan, United States; University of Michigan, Ann Arbor, Michigan, United States; Duke University, Durham, North Carolina, United States; University of Michigan, Ann Arbor, Michigan, United States

## Abstract

Research documents a positive association between high levels of neighborhood segregation and several negative health outcomes among Black Americans. However, few studies examine the relationship between neighborhood segregation and the health of both Black and White Americans. We apply a lifecourse framework to better understand how exposure to neighborhood segregation earlier in life may lead to racial inequities in health byway of cumulative advantages/disadvantages (CAD). Utilizing data from the nationally-representative Americans’ Changing Lives study (ACL), we employ growth curve models to investigate the relationship between neighborhood segregation in 1990 and the trajectories of two health outcomes over the adult lifespan: the number of chronic conditions and the probability of experiencing functional limitations. Our analysis reveals that, for Black participants, the baseline level of black segregation does not significantly impact their trajectories in these health outcomes. However, among White participants, residing in neighborhoods with lower levels of white segregation is associated with an increased probability of developing more chronic conditions and experiencing functional limitations later in life. We explore the roles of systemic and cultural racism as shaping forces in neighborhood structures and as contributors to negative health outcomes for both Black and White Americans. Research implications include further parsing different types of white underrepresentation in neighborhoods (i.e., living in predominantly Black neighborhoods vs racially diverse integrated areas) to examine their unique effects on health trajectories. Further, we discuss how intervention efforts may identify and address detractors from healthy aging for White Americans who are underrepresented in their neighborhoods.

